# Placebo Groups in Research on the Effectiveness of ABA Therapeutic Techniques

**DOI:** 10.3389/fpsyg.2018.01899

**Published:** 2018-10-18

**Authors:** Przemysław Bąbel, Elżbieta Anita Bajcar, Katarzyna Marchewka, Katarzyna Sikora

**Affiliations:** ^1^Pain Research Group, Institute of Psychology, Jagiellonian University, Kraków, Poland; ^2^Institute of Psychology, Jagiellonian University, Kraków, Poland; ^3^Institute of Philosophy, Jagiellonian University, Kraków, Poland; ^4^Institute of Psychology, Jesuit University Ignatianum, Kraków, Poland

**Keywords:** applied behavior analysis, effectiveness of psychotherapy, ethics, placebo, randomized placebo-controlled trials, single-subject experimental design

## Abstract

Behavior analysts have shown that a single-subject experimental design (SSED) is a useful tool for identifying the effectiveness of specific therapeutic techniques, whereas researchers outside applied behavior analysis (ABA) maintain that randomized placebo-controlled trials (RPCT) provide the most definite test of efficacy. In this paper the possible benefits that could result from supporting SSED studies by placebo control groups are discussed. However, the use of placebo groups in psychotherapy research arouses considerable controversy and many researchers argue against it. The main aim of this paper is to clarify theoretical and methodological problems associated with using placebo groups in psychotherapy research and to demonstrate that these problems can be solved if the assumptions on which they are based are reformulated. The article also discusses ethical issues about the use of placebo groups in research on the effectiveness of psychotherapy.

## Introduction

It is well known that different forms of psychotherapy can have similar levels of effectiveness (Luborsky et al., 1975, 2002; Smith and Glass, 1977; Shapiro and Shapiro, 1982; Wampold et al., 1997; Wampold and Imel, 2015). This effect is often called the Dodo Bird verdict from the famous paper by Rosenzweig (1936) that contains an epigraph from Lewis Caroll’s “Alice in Wonderland”: “At last the Dodo said, ‘Everybody has won, and all must have prizes’.” Based on this verdict, the conclusion is usually drawn that different types of psychotherapy are equally effective. However, because there are upward 500 of brand-name psychotherapies (Aveline, 2001) and over 500 different approaches to psychotherapy (Mozdzierz et al., 2014), it is senseless to pose the question of whether or not psychotherapy in general is effective.

Asking about the general effectiveness of psychotherapy is similar to asking about the general effectiveness of medicines or drugs. It is possible to assess the effectiveness of a specific medicine (e.g., paracetamol) for a specific condition (e.g., headache), but not the general effectiveness of different medicines for different conditions. Similarly, one can assess the effectiveness of a specific therapeutic technique (e.g., systematic desensitization) for a specific condition (e.g., a phobia), but not the general effectiveness of different forms of psychotherapy for different conditions. For example, there are at least 111 different treatments used for autism in children (Green et al., 2006), but only some of them have been empirically supported (National Autism Center, 2009, 2015; Wong et al., 2014). In other words, the effectiveness of different therapeutic techniques for specific conditions may vary substantially. Therefore, the question is not whether psychotherapy is effective, but whether a specific form of psychotherapy treatment is effective. However, it is still not clear what methods should be used to properly assess the effectiveness of various therapeutic interventions.

## The Use of Single-Subject Experimental Designs in Psychotherapy Research

Applied behavior analysis (ABA) “is the science in which tactics derived from the principles of behavior are applied systematically to improve socially significant behavior and experimentation is used to identify the variables responsible for behavior change” (Cooper et al., 2007, p. 20). ABA offers very specific therapeutic techniques that involve manipulating the antecedents and consequences of a behavior in order to change it. Most ABA techniques are based on positive and negative reinforcement, as well as positive and negative punishment (Cooper et al., 2007).

One may challenge the idea that ABA techniques are a form of psychotherapy. However, there are different definitions of psychotherapy, and the concept does not have to be restricted to talk therapy. Indeed, according to van Deth (2013) “psychotherapy is more than just talk therapy” (p. 6). Moreover, Norcross (1990) eclectic definition reads as follows: “Psychotherapy is the informed and intentional application of clinical methods and interpersonal stances derived from established psychological principles for the purpose of assisting people to modify their behaviors, cognitions, emotions, and/or other personal characteristics in directions that the participants deem desirable” (p. 218). In light of this definition, ABA techniques are a form of psychotherapy in that they are intentionally applied clinical methods derived from established psychological principles of behavior for the purpose of assisting people to modify their behaviors (as well as cognitions, emotions, and/or other personal characteristics which – according to radical behaviorism – are behaviors too) in desirable directions.

### Single-Subject Experimental Design

The essential component of ABA is a single-subject experimental design (SSED) (Bailey and Burch, 2002). This research design uses experimental techniques to establish casual relationships between a dependent variable (i.e., an observable target behavior) and an independent variable (i.e., a treatment). In order to obtain evidence that the treatment can change a target behavior, a participant is exposed to at least two different conditions – an experimental condition in which the independent variable is introduced and a control condition in which the independent variable is withdrawn. Therefore, the individual participant acts as his/her own control. The performance across conditions is observed and the efficacy of the intervention is inferred based on the results. Objective features of behavior (e.g., duration, intensity, and latency) are assessed by visual inspection. The findings are considered reliable if they are replicated. Although a SSED is often conducted with a single participant, it can also be administered to a group of individuals participating in the same study. Therefore, conclusions may be drawn by intra- and inter-participant replication (Bailey and Burch, 2002). This classic experimental design may be extended by multiple baseline design, in which baseline data are collected simultaneously across two or more subjects, settings, or behaviors and then the treatment is implemented sequentially across these subjects, settings or behaviors (Cooper et al., 2007). This form of experimental design allows to control for extraneous variables and analyze the effect of the treatment without its withdrawing. Multiple baseline design may be implemented in clinical settings in which treatment withdrawal is inadvisable or not possible because of ethical and practical reasons.

A key feature of a well-designed SSED is experimental control. The control condition should contain all the features of the experimental condition except for the independent variable. Thompson and Iwata (2005) reviewed various control procedures that are commonly used to examine the effectiveness of reinforcement, i.e., extinction, non-contingent reinforcement (NCR), differential reinforcement of another behavior (DRO), and differential reinforcement of an alternative behaviour (DRA). The most frequently used procedure is extinction, which occurs when reinforcement of previously reinforced behavior is discontinued. However, as Thompson and Iwata (2005) noted, this method has a serious limitation, in that the control condition does not contain all the characteristics of the experimental condition because not only is the contingency between the response and reinforcement eliminated, but the reinforcer is not presented. The three remaining procedures do not have this limitation. NCR involves the presentation of the reinforcer in the control condition according to a response-independent schedule, e.g., every 5 min. In DRO, the reinforcer is delivered contingent on the absence of the target-response, i.e., various behaviors may be reinforced, with the exception of those reinforced in the experimental condition. In DRA, a behavior is reinforced that is alternative to the behavior reinforced in the experimental condition.

Thompson and Iwata (2005) noted that although NCR is considered to be the most methodologically advantageous control procedure, it raises some concerns. Since reinforcement is applied independent of the target response (e.g., every 5 min) in the control condition, there is a risk that this response may be reinforced accidentally. Hence, the target response may be maintained during the control condition as a result of accidental reinforcement. Therefore, performance in the experimental and control conditions may not differ significantly, which may lead to the erroneous conclusion that the intervention applied in the experimental condition is ineffective or that its effect is weak.

Contingency reversal procedures (i.e., DRO and DRA) also have important limitations. As the reinforcement in both procedures is delivered during the control condition, the mere presentation of the previously established reinforcer may elicit the target response. As in the case of NCR, this can lead to erroneous conclusions regarding the effectiveness of the intervention applied in the experimental condition. Moreover, if this response is not reduced quickly, subsequent presentation of the reinforcer will be deferred. Thus, instead of applying contingency reversal procedures, extinction will be applied.

The difficulties discussed above derive from the fact that repeated measures on the same individual are not independent of one another. We believe that these limitations can be eliminated by using a different experimental design in which participants are assigned randomly to two separate groups and individuals do not serve as their own control. The additional use of randomized placebo-controlled trials (RPCTs) would allow one to verify the results obtained in SSED research and help to eliminate other objections to SSED results. Because SSED studies focus on the examination of individuals, their results may not be generalizable to other people across age, gender, and learning histories (Reboussin and Morgan, 1996). Results obtained in SSED research also fail to answer actuarial types of questions, e.g., what percentage of people who undergo treatment may benefit from it? and How many of them may respond negatively? Thus, a number of researchers maintain that RPCTs may provide the most definite test of efficacy and they call for using RPCTs as the most rigorous method to evaluate the efficacy of therapeutic interventions (Temple, 2002; Miller and Brody, 2003; Castro, 2007). Last but not least, as RPCTs are the so-called gold standard of studies on the effectiveness of both medical and psychotherapeutic treatments (Harrington, 2002), it is impossible to compare the results of the studies on the effectiveness of ABA techniques and other therapeutic techniques. In effect, the ABA approach is not as popular as it could be and its effectiveness and status as a psychotherapeutic approach are questionable (Todd and Morris, 1992; Chiesa, 2005). However, to apply an RPCT in researching the effectiveness of ABA techniques, it is necessary to define a placebo in the context of psychotherapy.

## The Use of Randomized Placebo-Controlled Trials in Psychotherapy Research

### Theoretical Considerations

The placebo in psychotherapy is often defined in terms of common factors. Critelli and Neumann (1984) concluded that “the common-factors criterion appears to be the most viable current definition of the placebo for the study of psychotherapy” (p. 35). However, there are at least three problems in the common factors perspective on the use of placebo groups in psychotherapy research. First, are common factors really common? This seems unlikely given that Grencavage and Norcross (1990) identified a total of 89 different common factors! Second, one of the common factors, i.e., the therapeutic relationship, seems to be crucial. Norcross and Wampold (2011) concluded that “the therapy relationship makes substantial and consistent contributions to psychotherapy outcomes independent of the specific type of treatment. The therapy relationship accounts for why clients improve (or fail to improve) at least as much as the particular treatment method” (p. 98). However, is the therapeutic relationship really so crucial regardless of the specific type of treatment? Are there any forms of psychotherapy in which the therapeutic relationship barely plays a role? Third, as discussed above, different forms of psychotherapy appear to be equally effective (Luborsky et al., 1975, 2002; Smith and Glass, 1977; Shapiro and Shapiro, 1982; Wampold et al., 1997; Wampold and Imel, 2015). The reason for the Dodo Bird verdict is often found in the assumption that the effectiveness of all forms of psychotherapy result from common factors. If so, psychotherapy is, in fact, a placebo. Moreover, if the effectiveness of all psychotherapies relies only on common factors, it is impossible to identify their ‘active’ ingredients. It is not, therefore, sensible to speak of constructing a placebo in psychotherapy.

However, according to Grünbaum’s (1981, 1985, 1986 see also Howick, 2017) concept of a placebo, “whether a given positive effect on D [target disorder] is or is not a placebo effect depends on whether it is produced by the incidental treatment factors or the characteristic ones” (Grünbaum, 1985, p. 15). In light of this conceptualisation, common factors may be either incidental or characteristic treatment factors, depending on the specific type of the psychotherapy and the therapeutic technique in question. In other words, in some cases a specific common factor (e.g., talking with the client) may be a placebo (an incidental treatment factor), but in other instances it may be an active ingredient (a characteristic treatment factor). More generally, the same active ingredient of psychotherapy may be either an incidental or a characteristic treatment factor. Using Grünbaum’s (1981, 1985, 1986) conceptualisation, it is possible to adequately define placebo within psychotherapy as a *therapeutic technique that only uses incidental treatment factors*.

RPCT should be distinguished from other types of comparative trials, like those which use ‘inert’ controls, i.e., a wait-list control or no treatment control group (Mohr et al., 2009). Unlike the placebo group, the participants in those two groups do *not* receive any treatment during the study. Those in a wait-list control group are informed that they will receive treatment after the treatment group has received it. It should be emphasized that using wait-list control and no treatment groups does not allow the control of the aforementioned incidental treatment factors. They may be controlled only if placebo groups are implemented. Therefore, the use of placebo groups contributes to the credibility of the obtained results. However, the question is how to create placebo groups that contain incidental, but not characteristic treatment factors.

### Methodological Considerations

There are two major methodological questions associated with the use of placebo groups in psychotherapy research: (1) whether the use of a placebo group in research is fully justified, and (2) whether it is possible to create placebo groups in psychotherapy research.

Some authors argue against the use of placebo groups in psychotherapy research and recommend comparing a new treatment to one that is evidence-based, i.e., its efficacy has been proven already (Rothman and Michels, 1994, 2002; Freedman et al., 1996). However, this proposal is based on ethical rather than on methodological grounds. In many cases, comparing a new treatment with an established one can bring inconclusive results, since clear conclusions can be drawn only if the new treatment works better than the established one. However, if the new treatment is less effective than the control treatment, it is impossible to assess whether the new treatment is simply less effective than the control treatment, or whether the new treatment is not effective at all. In order to have an objective assessment in such cases, the new treatment needs to be compared with a placebo treatment. Even if the new treatment is determined to be less effective than the established one, it is still worth using a placebo control group to verify whether it is effective at all. If it is, then it may be applied in conditions where the standard treatment cannot be used (Temple, 2002). Thus, it seems that at least in some cases the use of a placebo group is necessary to obtain significant and credible results (Ellenberg and Temple, 2000; Pocock, 2002; Temple, 2002; Millum and Grady, 2013).

The use of placebo groups in the context of ABA can lead to at least four benefits: (1) it will demonstrate that creating placebo groups is possible in research on the effectiveness of psychotherapy; (2) the quality of research on the effectiveness of psychotherapy will be improved; (3) it may help to disseminate and promote the ABA approach by making it possible to compare the effectiveness of ABA techniques and other therapeutic techniques; and (4) it may demonstrate that ABA techniques are not only applicable to the treatment of autism, but are a scientific approach with a wide range of applications. If the last two of these goals are achieved, Bailey’s objective to redefine and strengthen the position of ABA in contemporary culture will be realized (Bailey, 2000).

We propose the ways of creating placebo interventions in research on the effectiveness of ABA techniques by the use of a specific, ‘active’ ABA technique that is not functionally relevant for the behavior being treated. Examples of using such ABA techniques are presented below^1^.

#### Functionally Irrelevant Use of ABA Techniques

If a specific ABA technique is used to reinforce a target behavior (e.g., a child gets a token after every 5 min of being silent; this is a token economy technique, see Cooper et al., 2007), a DRO procedure may be used in a placebo group to reinforce a non-target behavior (e.g., a child gets a token every 5 min when she is not silent, although the target behavior is being silent). Alternatively, one could use NCR to reinforce a target behavior (e.g., a child gets a token every 5 min regardless of what she does, although the target behavior is being silent), or promises of a reinforcer (e.g., a child is promised a token after every 5 min of being silent and the promise is not kept). The DRA procedure can also be applied in a placebo group (e.g., a child gets a token when she makes noise, although the target behavior is being silent).

If a specific ABA technique uses the punishment of a problem behavior (e.g., every time a child hits someone, she is required to stand up and sit down 10 times; this is a contingent exercise technique; see Cooper et al., 2007), the technique in the placebo group could be intermittent punishment (e.g., every third instance of a child hitting someone, she is required to stand up and sit down 10 times), or interruption of punishment before the problem behavior ceases (e.g., every time a child hits someone, she is required to stand up and sit down 10 times; however, this procedure is applied only until the 6th appearance of the problem behavior), or threats of punishers (e.g., every time a child hits someone, the child is told that the next time she will be required to stand up and sit down 10 times, but the punishment never happens). One of the main principles of effective punishment is to use punishers every time the target behavior is emitted. If this is not done, the target behavior is negatively reinforced and its frequency increases rather than decreases. This is why intermittent punishment and interruption of punishment are placebos when punishment is used as a nonplacebo (‘active’) intervention.

When one of the extinction procedures is used as a specific ABA technique (e.g., when a child refuses food, the spoon is held to his/her mouth until she takes a bite; this is an escape extinction technique; Cooper et al., 2007), the technique in the placebo group could be the interruption of extinction before the problem behavior ceases (e.g., when a child refuses food, the spoon is held to his/her mouth until she takes a bite, however, this procedure is applied only 10 times). One of the main principles of extinction is to continue it unless the target behavior has decreased. If this is not the case, the target behavior is reinforced and its frequency increases rather than decreases. That is why the interruption of extinction is a placebo when extinction is used as a nonplacebo (active), intervention.

The placebo interventions suggested above are at variance with the principles of behavior (Cooper et al., 2007). If the proposed placebos work, it could mean that the placebo response has occurred, i.e., the effect was produced by the incidental rather than by characteristic therapeutic factors. The application of the aforementioned control procedures in a placebo group enables one to avoid the limitations described in the previous section of the paper.

In the case of ABA techniques used to reduce problem behavior, one can refer to the function of the behavior to create a placebo group. The function of a behavior is an environmental stimulus that maintains a given behavior, i.e., reinforcing the behavior (Umbreit et al., 2006). To choose an ABA technique to reduce a problem behavior, one needs to find the function of that behavior. Functional assessment is a set of procedures to identify the functions of a problem behavior (Cooper et al., 2007). For example, if the function of the problem behavior is attention, one of the techniques that can be applied is extinction – the behavior does not produce attention, e.g., when a child twirls a plate on a table to get his/her father’s attention, the father ignores the behavior. However, if the function of the problem behavior is sensory stimulation, one of the techniques that can be applied is another form of extinction – sensory extinction, in which a sensory consequence is masked or removed, e.g., when a child twirls a plate on a table to produce auditory stimulation, one can cover the surface of the table. We propose that an ABA technique that is the opposite of the technique proposed on the basis of the results of a functional assessment of a problem behavior may serve as a placebo intervention. In the example discussed above, sensory extinction can be an ‘active’ technique applied to the problem behavior maintained by sensory stimulation, and ignoring the behavior can be a placebo in that case. If ignoring is successful, it means that the placebo response has occurred. This proposal is similar to an active placebo used in clinical trials on the effectiveness of medical treatments, because an ABA technique brings no specific result in the given case. If the functional assessment was conducted appropriately, and an ABA technique that was not based on the results of the functional assessment (i.e., placebo) had an effect on the problem behavior, it would be due to the effect of incidental rather than characteristic treatment factors. In this way, placebo groups may help to establish the mechanisms of ABA techniques.

To summarize, as the effectiveness of ABA techniques rarely relies on incidental therapeutic factors alone, and one can identify their ‘active’ (characteristic) ingredients, it is possible to create placebo interventions and use them in placebo groups in research on the effectiveness of psychotherapy, i.e., ABA techniques. Most importantly, the proposed placebo interventions should be credible because they are ‘active’ techniques, but they should be functionally irrelevant for the behavior being treated (see **Table 1** for summary of the proposed placebo interventions). They are placebos because they cannot be effective as a result of their own ‘action’. If they work, their results will be placebo effects. It should be noted that even though the proposed placebo interventions are intended to be credible, their features are not identical to the features of ‘active’ interventions, as is the case in medical research, where the color, shape, label, etc., of a placebo medicine and an active medicine are identical. We do not think that creating identical placebo and ‘active’ interventions in psychotherapy research is possible, but we are convinced that credibility is both necessary and sufficient to use placebo interventions in research on the effectiveness of psychotherapeutic techniques.

**Table 1:** Examples of the implementation of applied behavior analysis (ABA) techniques in experimental and placebo groups.

Aim of intervention	Experimental group (appropriate techniques that affect target behavior)	Placebo group (functionally irrelevant techniques)
Increasing the frequency of behavior	Continuous reinforcement of target behavior (CRF)	Differential reinforcement of other behavior (DRO)
Increasing the frequency of behavior	Non-contingent reinforcement of target behavior (NCR)	Differential reinforcement of alternative behavior (DRA)
Decreasing the frequency of behavior	Continuous punishment	Intermittent punishment of target behavior
Decreasing the frequency of behavior	Continuous punishment	Interruption of punishment before the target behavior ceases


Although it is both possible and desirable to create placebo groups in research on the effectiveness of ABA techniques, some say the use placebo groups should be avoided for ethical reasons (Rothman and Michels, 1994, 2002; Freedman et al., 1996). Thus, one question is whether using placebo groups in research on psychotherapy in general, and in research on ABA techniques in particular, is ethical.

### Ethical Considerations

The use of RPCTs in clinical research is considered risky for subjects in a placebo group because withholding an effective intervention can result in the deterioration of their condition. Therefore, placebo opponents claim that the use of a placebo should be permitted in research only if there is no procedure that can replace a placebo (Beauchamp and Childress, 2013). They contend that all patients should receive ‘the best proven current treatment,’ and they should not be exposed to even minor discomfort. For that reason, some ethicists and researchers recommend using active control trials that compare a new treatment with a treatment known to be effective (Rothman and Michels, 1994, 2002; Freedman et al., 1996). However, the use of active control trials raises some methodological and interpretative problems, as discussed above, and it does not reduce the ethical concerns because those who undergo the new treatment instead of the established one do not receive ‘the best proven current treatment’. Furthermore, trials with an active control may expose more people to harm than an RPCT because the former requires a larger sample size in order to achieve sufficient power (Temple and Ellenberg, 2000).

The complaint that using placebo groups in psychotherapy research deprives participants of treatment is not fully justified in the context of the fundamental bioethical distinction between research and practice (The Belmont Report, 2018). Clinical practice is an activity aimed at improving the medical welfare of the patient, whereas research focuses on an examination of a research hypothesis. Different purposes have different ethical considerations associated with them. Hence, a person who agrees to participate in an experiment must already know that the selection of the research group (and, therefore, the placebo group) is random. Thus, the person should realize that she may be in a group that is not the most suitable, given his/her health. The duty of researchers is to do everything they can to make participants aware that in an experimental situation a therapist is a researcher and his/her aim is scientific truth. It must be stressed, however, that exposing participants to a placebo treatment can only be accepted if the knowledge resulting from the studies will be used to refine common therapeutic techniques and eliminate inefficient, costly, and potentially harmful therapies. In other words, the risks for research subjects posed by their participation in a research project should be justified by the anticipated benefits for the subjects and/or for society.

A study is not feasible in a situation where the benefit of the research requires a delay in providing information about the use of a placebo. Beauchamp and Childress (2013) suggested that researchers should inform participants that a placebo will be used but should not give specific information; for example when and how it will be used. However, according to the criteria for obtaining informed consent (Beauchamp and Childress, 2013), if a participant does not receive sufficient information about a treatment (e.g., if the participant does not know exactly when the placebo will be used during the experiment), she cannot give informed and voluntary consent to participate in the research. Bok (2002) stressed that participants must be asked for consent for using a placebo even if they take part in a double-blind study, and they will not know if they are in a group receiving a placebo or an active treatment. To conclude, participants must be informed that a placebo may be used in the research and the probability of their inclusion in a placebo control group is 50%; however, there is no need to give specific information about the placebo, e.g., who will receive it. Participants must also be informed about the possible benefits and risks from receiving or not receiving treatment in order to give fully informed consent (Schafer, 1982).

Some investigators and theorists are convinced that people, even those who are well-informed, are unable to judge the experimental situation and to make a conscious decision concerning their participation in a study because of their lack of expertise. Thus, they propose that studies should not include placebo groups (Rothman and Michels, 1994, 2002). However, as Temple (2002) noted, such an attitude toward participation in an RPCT undermines the right of every person to decide on their own to participate or not. On the other hand, there are specific groups of people who are not able to decide and to give voluntary consent because of their age (e.g., small children, the elderly), their health status (e.g., psychiatric patients, mentally handicapped persons), or other reasons. Proxy consent is required in such cases, but this raises another ethical question, of whether proxies are eligible to provide consent on their own behalf. Regardless of this concern, there is general agreement that those people who are particularly vulnerable to harm should be particularly protected against being exploited as research subjects (Bok, 2002). Nevertheless, their exclusion from studies may hinder or delay the development of effective therapies that could improve their functioning.

## Conclusions

Both SSEDs and RPCTs are not necessarily incompatible and they may even be considered to be complementary. An SSED is advantageous because it allows the evaluation of the effectiveness of a therapeutic intervention with an individual. Because an SSED study examines the behavior of a particular person in a particular situation, it is ideal for clinical application (Kazdin, 2010). It provides immediate feedback and enables the verification of an applied treatment and the adaptation of interventions to the client’s needs. Because SSED studies accurately document the effectiveness of therapeutic interventions, their results may be used as pilot data for RPCTs.

The results of SSED studies undoubtedly help expand knowledge of the effectiveness of therapeutic interventions (Horner et al., 2005; Kazdin, 2010; Kratochwill and Levin, 2010; Byiers et al., 2012). However, this methodological approach has a number of drawbacks that may affect its results (Long and Hollin, 1995; Newcombe, 2005; Alnahdi, 2015). Supporting SSED results by using an RPCT could help to eliminate or reduce those limitations. Moreover, by using techniques that are specific to SSEDs in RPCTs, it is possible to compare the results obtained in SSED research with those derived from an RPCT. Using an RPCT in research on the effectiveness of ABA techniques also may help to disseminate and promote the ABA approach by making it possible to compare the effectiveness of ABA techniques with those of other therapeutic techniques.

In this paper, we attempted to show that the use of placebo groups in research on the effectiveness of ABA techniques is possible practically, theoretically, and methodologically. However, it may be problematic from an ethical point of view. Two main types of ethical dilemma emerge when considering the use of a placebo in psychotherapy research, both of which regard potential harm: first to the client, and second to society. The controversies of the first type refer to violation of the client’s autonomy by applying ineffective treatment and losing the client’s trust. Conducting an RPCT means exposing participants to a prolonged duration of treatment with only incidental therapeutic factors, while withholding another treatment that might be provided through contact with a benevolent person. The use of placebo interventions may not only subject a person to prolonged treatment with incidental factors, it may also maintain or enhance problem behaviors temporarily. However, it is possible to avoid some unwanted consequences or minimize the harm associated with the use of placebo groups by implementing standard precautions for an RPCT. Above all, participants must be informed that a placebo control group is used in the study and they must be aware that they will be assigned randomly to the treatment groups. The participants must be debriefed at the completion of the study. They must know that they can refuse to participate at any point during the study and be assured that they will be treated with evidence-based therapy outside the trial. Participants should be carefully monitored during clinical research and those who develop serious symptoms should be removed from a clinical trial and provided with effective treatment. The placebo treatment should be limited to the minimum time required for the results to be scientifically valid (Emanuel and Miller, 2001). The placebo control group participants should undergo the treatment at the completion of the study to eliminate undesirable reactions, i.e., a target intervention may be used.

Controversies of the second kind (harm to society) refer mainly to the social costs of *not* conducting placebo-controlled psychotherapy research, and involve the question of whether psychotherapy has a place in a public healthcare system, if its effectiveness is not assessed or it is shown to be based solely on incidental therapeutic factors. Undoubtedly, research using placebo groups can be used to provide knowledge useful for the whole society and to obtain useful results more easily. RPCTs may have some advantages over active control trials or SSED studies. Active-control trials require more participants than RPCTs to establish sufficient power and they take much more time (Millum and Grady, 2013). Small sample size which is typical to the SSED studies also limits generalizability of the results to other groups or settings. Moreover, repeatedly administered assessment is required to obtain valid results. It seems that including the RPCTs to the studies on the effectiveness of therapeutic techniques might help to overcome some of these limitations. The use of RPCTs may be particularly advantageous in the case of low-prevalence disorders, because it can be difficult to recruit a large number of subjects (Millum and Grady, 2013). Therefore, if there is no threat to life and no risk of permanent and irreversible negative consequences, the use of placebo control groups should be carefully considered.

## Author Contributions

PB conceptualized the manuscript and drafted the introduction and the section entitled ‘The use of randomized placebo-controlled trials in psychotherapy research’. KM and KS drafted the section ‘Ethical considerations’. All the authors drafted the conclusions. EB drafted the section entitled “The use of single-subject experimental designs in psychotherapy research’ and revised the manuscript.

## Conflict of Interest Statement

The authors declare that the research was conducted in the absence of any commercial or financial relationships that could be construed as a potential conflict of interest.

## Footnotes

1 Although our examples of applied behaviour analysis (ABA) techniques deal with children’s behaviors, ABA is a very broad scientific discipline and its techniques may be applied in many different areas, not only in education (cf. Vollmer et al., 2000). In practice, ABA techniques are not used in isolation as they are in our examples. However, using a specific technique in isolation may be necessary in research on its effectiveness.
